# Lipidated Polyaza Crown Ethers as Membrane Anchors for DNA-Controlled Content Mixing between Liposomes

**DOI:** 10.1038/s41598-019-49862-y

**Published:** 2019-09-25

**Authors:** Philipp M. G. Löffler, Anders Højgaard Hansen, Oliver Ries, Ulla Jakobsen, Alexander Rabe, Kristian T. Sørensen, Kasper Glud, Stefan Vogel

**Affiliations:** 1 Department of Physics, Chemistry and Pharmacy, University of Southern Denmark, Campusvej 55, DK-5230 Odense M, Denmark University of Southern Denmark, Odense M, Denmark; 2 PET & Cyclotron Unit, Department of Nuclear Medicine, Odense University Hospital, Sdr. Boulevard 29, 5000 Odense C, Denmark

**Keywords:** Membrane biophysics, Membrane structure and assembly, Origin of life

## Abstract

The ability to manipulate and fuse nano-compartmentalized volumes addresses a demand for spatiotemporal control in the field of synthetic biology, for example in the bottom-up construction of (bio)chemical nanoreactors and for the interrogation of enzymatic reactions in confined space. Herein, we mix entrapped sub-attoliter volumes of liposomes (~135 nm diameter) via lipid bilayer fusion, facilitated by the hybridization of membrane-anchored lipidated oligonucleotides. We report on an improved synthesis of the membrane-anchor phosphoramidites that allows for a flexible choice of lipophilic moiety. Lipid-nucleic acid conjugates (LiNAs) with and without triethylene glycol spacers between anchor and the 17 nt binding sequence were synthesized and their fusogenic potential evaluated. A fluorescence-based content mixing assay was employed for kinetic monitoring of fusion of the bulk liposome populations at different temperatures. Data obtained at 50 °C indicated a quantitative conversion of the limiting liposome population into fused liposomes and an unprecedently high initial fusion rate was observed. For most conditions and designs only low leakage during fusion was observed. These results consolidate LiNA-mediated membrane fusion as a robust platform for programming compartmentalized chemical and enzymatic reactions.

## Introduction

Conjugation of oligonucleotides with neutral lipids (lipid-nucleic acid conjugates, LiNAs) has proven a viable strategy for functionalization of artificial lipid membranes^1,2^. Synthetic lipid-based membranes and liposomes represent useful model systems for studying membrane docking and fusion, and serve as platforms for nano-scaffolded (bio)chemical processes, e.g. reactions catalyzed by immobilized or compartmentalized enzymes, including a variety of other applications revolving around efficient spatiotemporal manipulation of sub-attoliter compartments.

In recent years, techniques involving LiNAs for controlled aggregation^3–6^ and fusion^7–10^ of phospholipid-based liposomes have been developed, but only recently, substantial content mixing (CM) between LiNA-encoded liposome populations has been reported (i.e. ≥20% of encapsulated volume)^11,12^. To use DNA as a tool for programming multiple rounds of fusion, efficiencies above 20% are needed to achieve detectable fusion beyond the first round. Recently, the feature of programmability in LiNA-mediated fusion was demonstrated in experiments where liposomes were fused over three sequentially dependent rounds, based on the design of the nucleobase sequences of the LiNA fusogens. An estimated bulk fusion yield of 60-80% per round was reported^13^.

The most well-described proteins that drive biological fusion are the soluble *N*-ethylmaleimide-sensitive-factor attachment receptors (SNAREs). SNAREs force biological membranes into contact through the interaction of membrane-anchored subunits. Compared to the LiNAs mentioned above, they also rely on transmembrane domains (TMDs) and a conserved linker-domain for efficient fusion^14–16^. SNARE-model systems such as SNARE sub-domains (SNAREpins)^17^ and SNARE-derived lipidated coiled-coil peptides^18,19^ were also shown to induce fusion and content mixing of liposomes. Using nucleic acid hybridization as a recognition unit provides means to make liposome fusion programmable, making use of Watson-Crick base pairing to achieve specific recognition and fusion between multiple populations of liposomes. In Biology, SNAREs rely on a variety of co-factors for specificity, and coiled-coil forming peptides lack the intrinsic programmability of nucleic acids^16,20^.

Further model systems include peptide nucleic acids (PNA) conjugated to the SNARE TMD-peptides, which were used to study the role of TMDs in fusion pore opening^20–22^. Recently PNAs with aliphatic membrane anchors were employed^23^, serving as a useful uncharged alternative to LiNAs. Many of the earlier fusion systems have been reviewed in the literature showing either low levels of content mixing or very substantial leakage for designs with higher levels of content mixing^24,25^.

Compared to SNARE-derived lipidated coiled-coil peptides, only a low degree of functionalization of LiNAs is needed to facilitate efficient content mixing between liposomes (~0.5 to 5 mol% versus 0.01 to 0.1 mol% of total lipid for lipopeptides and LiNAs, respectively)^18,19^. Still, it was estimated that the number of LiNA duplexes per liposome required to open a fusion pore is around 10–20^9,13^, while native SNAREs are effective at 1-2 complexes per vesicle^26^.

**Figure 1:**
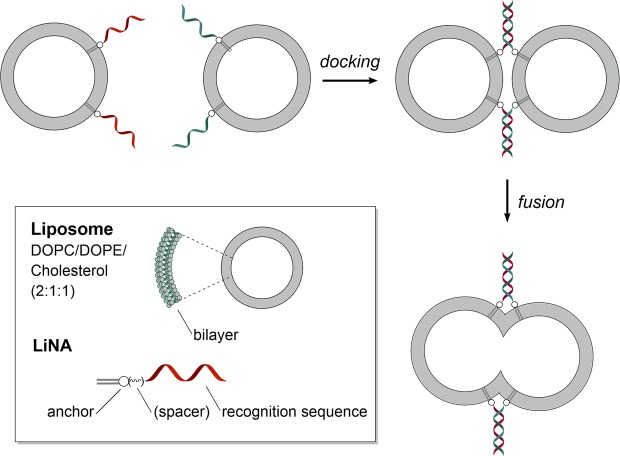
LiNA-mediated fusion of liposomes. Functionalized liposomes with complementary LiNAs dock via hybridization of LiNA strands leading to membrane fusion (drawing not to scale).

Regarding the mechanism of membrane fusion, SNARE-proteins act via coiled-coil formation between different α-helical subunits, and via local destabilization of the cell membrane induced via the TMDs and linker-domains of the SNARE-complex^20–22^. Concerning LiNAs or other SNARE-mimics lacking a TMD, we earlier proposed the hypothesis^12^ that the main driver for fusion are Brownian collisions between bilayers in tight apposition (docked state, Fig. 1). Spontaneous fusion requires intermembrane distances of ≤2 nm, at which the hydration shell of the lipid headgroups begins to be disturbed^27,28^. Notably, the rate of spontaneous membrane fusion, as measured by François-Martin *et al*.^28,29^, could simply be amplified in a docked state forcing intermembrane distances of a few nanometers across an extended contact area.

Applications of controlled and/or programmable liposome fusion have already been reported. Direct fusion to cell-membranes present a promising strategy for bypassing endosomal pathways during drug delivery. Lipidated SNARE-peptides have been used to fuse liposomal vehicles to live HeLa cells in culture (loaded with small molecules^30^ or oligonucleotides^31^) or even to endothelial cells in the blood stream of live zebrafish embryos^32^. Just recently, Sun *et al*. reported on DNA-mediated fusion to deliver horseradish peroxidase directly to the cytosol of L1210 and HeLa cells as a model for protein delivery^33^.

**Figure 2:**
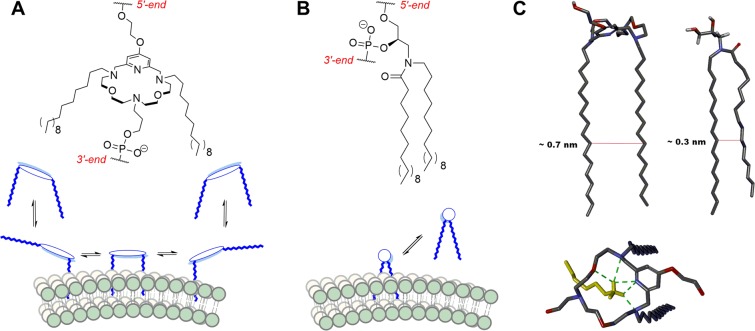
Anchor design. Different anchor scaffold structures and proposed modes of anchoring (**A**) Anchor based on aza-crown ether scaffold has a 6-bond spacing over a rigid aromatic system giving the two lipophilic chains an approximate spacing of 0.7 nm and allowing each chain to interact with a different subset of lipids in the bilayer. (**B**) In the anchor based on 3-amino-1,2-propanediol scaffold both chains are attached to the same atom, favoring intramolecular interactions of the two chains, effectively resulting in a bigger lipophilic moiety which is expected to interact with the membrane in a concerted manner (**C**) Molecular representation of average lipid chain distances (red lines) for relaxed palmityl chains in aza crown ether and amino propanediol membrane anchors (hydrogens not shown for clarity) and top view of the aza crown ether macrocycle with hydrogen bonded (green dotted lines) ammonium head group of aminoethanol lipids from the lipid bilayer.

In our efforts to develop a robust platform for sequence-programmable membrane fusion to be applied in systems biochemistry and as nanoreactors, we have previously studied the effect of different anchor moieties on fusion efficiency, i.e. two linear (palmityl), branched (phytanyl) or planar (cholesteryl) moieties were attached to the same N-atom of a 3-amino-1,2-propanediol scaffold^34^. In the present study, we used a macrocyclic polyaza crown ether scaffold to give an approximate  0.7 nm spacing between the two aliphatic chains (C_16_, palmityl) serving as anchors (see Fig. 2). When attached to the crown-ether scaffold, the lipophilic chains can act as two separate membrane anchor moieties, which was proposed to improve anchoring of the oligonucleotides compared to the case where both C_16_ chains are attached to the same N-atom. Furthermore, crown-ethers are known for their ability to bind cations. Aza-crown ethers differ from their oxo counterparts by their selectivity shift towards NH_4_^+^ over K^+^ and Na^+^ ions, when containing more than two N-atoms. For triaza crown ethers the affinity for binding ammonium ions was highest among the measured monovalent cations, in solution^35^ and as part of an oligonucleotide backbone^36^, respectively. As lipid mixtures used for membrane fusion models typically contain phosphatidylethanolamine (PE) lipids, the crown-ether may further enhance anchoring by binding to PE headgroups (present as zwitter-ions at neutral pH, with the ammonium moiety closest to the membrane surface)^36^. Here, we report that LiNAs carrying this macrocyclic non-nucleosidic building block efficiently mediate content mixing between two liposome populations engrafted with complementary LiNA strands.

## Results and Discussion

### Synthesis of double-chain anchor building blocks for solid-phase DNA synthesis

**Figure 3:**
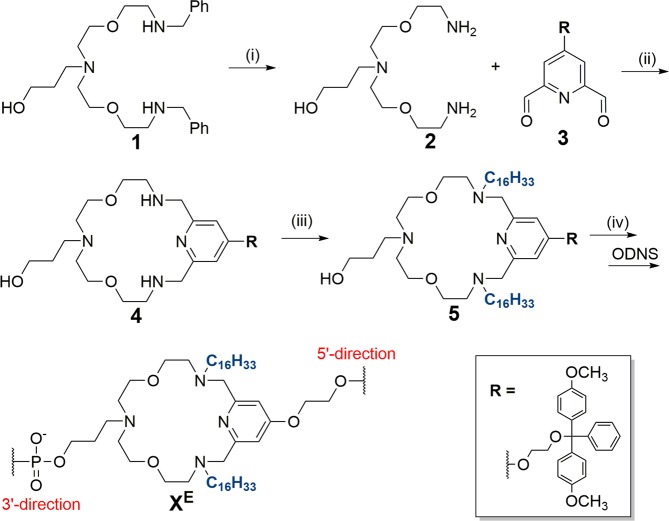
Synthesis and reaction conditions for compound 4-5a. Synthesis of **1** and **3** are described in ref.^37^. (i) Pd(OH)_2_, MeOH, rt (**2**, 90%). (ii) 1: DMTr protected dialdehyde, CaCl_2_, anhydrous MeOH, 3Å mol. sieves, reflux; 2: NaBH_4_, 5 °C → rt (**4**, 59%). (iii) 1: Hexadecanal, 2: NaBH(OAc)_3_, anhydrous DCE, 5 °C → rt, 3Å mol. sieves (**5**, 50%). (iv) 2-Cyanoethyl-*N*,*N*-diisopropylchlorophosphoramidite, anhydrous DCE, 5 °C → rt (**5a**, 80%). **5a** incorporated of into oligonucleotides = X^E^_._

**Figure 4:**
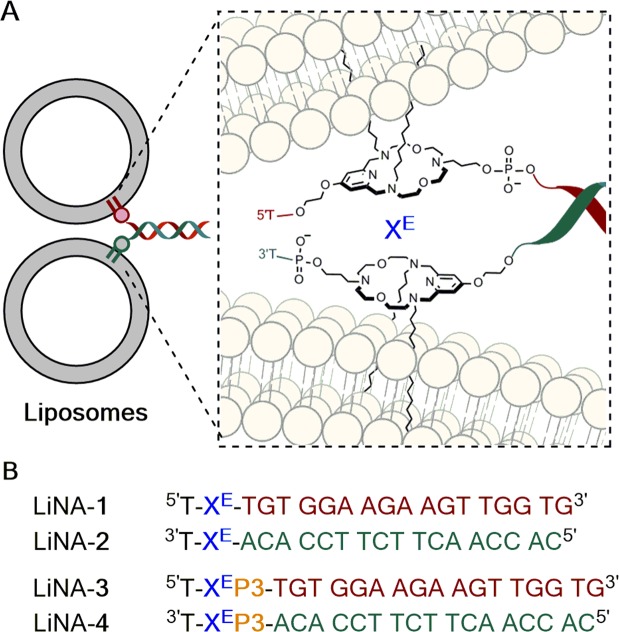
LiNAs for liposome fusion. (**A**) Schematic of liposome docking via hybridization of LiNA strands. The inset shows anchor structure and connectivity (drawing not to scale). (**B**) LiNA sequences with or without triethylene glycol phosphate spacer unit (P3). Duplexes between LiNAs complementary reference DNAs, i.e. the 17-nt zipper-sequence alone, have a *T*_m_ of around 57 ± 1 °C (see Supplementary information, Table S2).

We have previously reported on a lipid functionalized macrocyclic anchor building block that was inserted into DNA oligomers to facilitate DNA-mediated assembly of liposomes^3^. Rohr *et al*. described an early-stage insertion of lipid moieties into the polyaza crown ether scaffold, i.e., at the level of compound **1** (Fig. 3). Herein, we report on an improved synthetic strategy that allows for later-stage lipid anchor functionalization. The syntheses of benzylated diamine **1** and dialdehyde **3** have been described previously^37^. Hence, starting from **1** (see Fig. 3), the final phosphoramidite **5a** was obtained over four steps. First, treating **1** with palladium hydroxide in methanol at room temperature provided the deprotected diamine **2**. Next, reacting **2** with dialdehyde **3** in a standard reductive amination under dry conditions afforded the desired cyclized polyaza crown ether **4**, which was subsequently condensed with hexadecanal to give **5**. Finally, phosphitylation of **5** provided the desired lipid anchor building block **5a** primed for standard automated solid-phase oligonucleotide synthesis of LiNAs 1–4 (Fig. 4).

### Design, synthesis and characterization of LiNA fusogens

Previous studies used a design of DNA oligomers that are able to fold up into a zipper-like geometry (Fig. 4) giving rise to a close distance between bilayers upon hybridization^8,10,34^. In this study we synthesized two pairs of modified DNA oligonucleotides that hybridize in the same manner. The first pair contained a single T overhang and one insertion of the **5a** building block within the phosphodiester backbone (designated X^E^) at the 5′ or 3′ end, respectively (LiNA-1 and LiNA-2). The single nucleotide overhang was introduced as in previous studies^3,4^ to prevent the self-aggregation of LiNA molecules into micelles in aqueous phase, driven by hydrophobic interactions between the anchors^38^. In presence of the extra nucleotide, two negatively charged phosphate groups flank the anchor building block electrostatically and sterically disfavoring micelle formation (absence of foaming of aqueous LiNA stock solutions). The second set contained a triethylene glycol phosphate spacer (P3, see Fig. 4) inserted in between the anchor X^E^ and the DNA-based zipper unit adding approximately 3 nm (1.5 nm for each spacer in the duplex, similar to the ones described for coiled-coil fusogens by Daudey *et al*.)^39^ to the maximal separation between docked bilayers (LiNA-3 and LiNA-4). Previously, using LiNAs based on the 3-amino-1,2-propanediol backbone, the presence of a P3 spacer increased fusion efficiency^12^. In these LiNAs the distance between the 5′-OH and the N-atom bearing the aliphatic anchors was only four bonds, compared to the nine bonds in the present design (X^E^ anchor). We hypothesized that relative to LiNA-1 and LiNA-2, which do not contain spacer P3, less strain-release would be expected upon liposomal fusion mediated by LiNA-3 and LiNA-4. In this study, these two pairs serve to evaluate the effect of linker size and flexibility on LiNA-mediated fusion of liposomes.

The oligonucleotides listed in Fig. 4B were purified on a reverse phase HPLC as previously described^34^. Circular dichroism spectroscopy revealed that both pairs (LiNA-1/2 and LiNA-3/4, Fig. 4B) formed standard B-type DNA duplexes (see the Supplementary Information). Thermal denaturation and circular dichroism experiments of the complementary strands confirmed that each X^E^ or X^E^-P3-modified DNA could bind to unmodified complementary sequences (Supplementary Information, Table S2 and Figure S1 and S2). The *T*_m_ of such duplexes (56 to 58 °C) were comparable to the unmodified reference duplex (55 °C), however, duplexes of LiNA-1/2 and LiNA-3/4 exhibited an increased *T*_m_ (77 ± 1 °C). This increase was attributed to the hydrophobic interactions between aligned alkyl chains. In the presence of liposomes, the lipid anchors are already embedded into the nonpolar environment of different phospholipid bilayers and cannot exhibit the abovementioned stabilization. When carrying out the fusion experiments at 50 °C, we argue that this is approx. 6-8 °C below the actual *T*_m_ of the system. No fusion above the *T*_m_ of the LiNA/DNA duplexes was observed at 60 °C (data not shown).

### LiNA-Liposome binding using Surface Plasmon Resonance (SPR)

**Figure 5:**
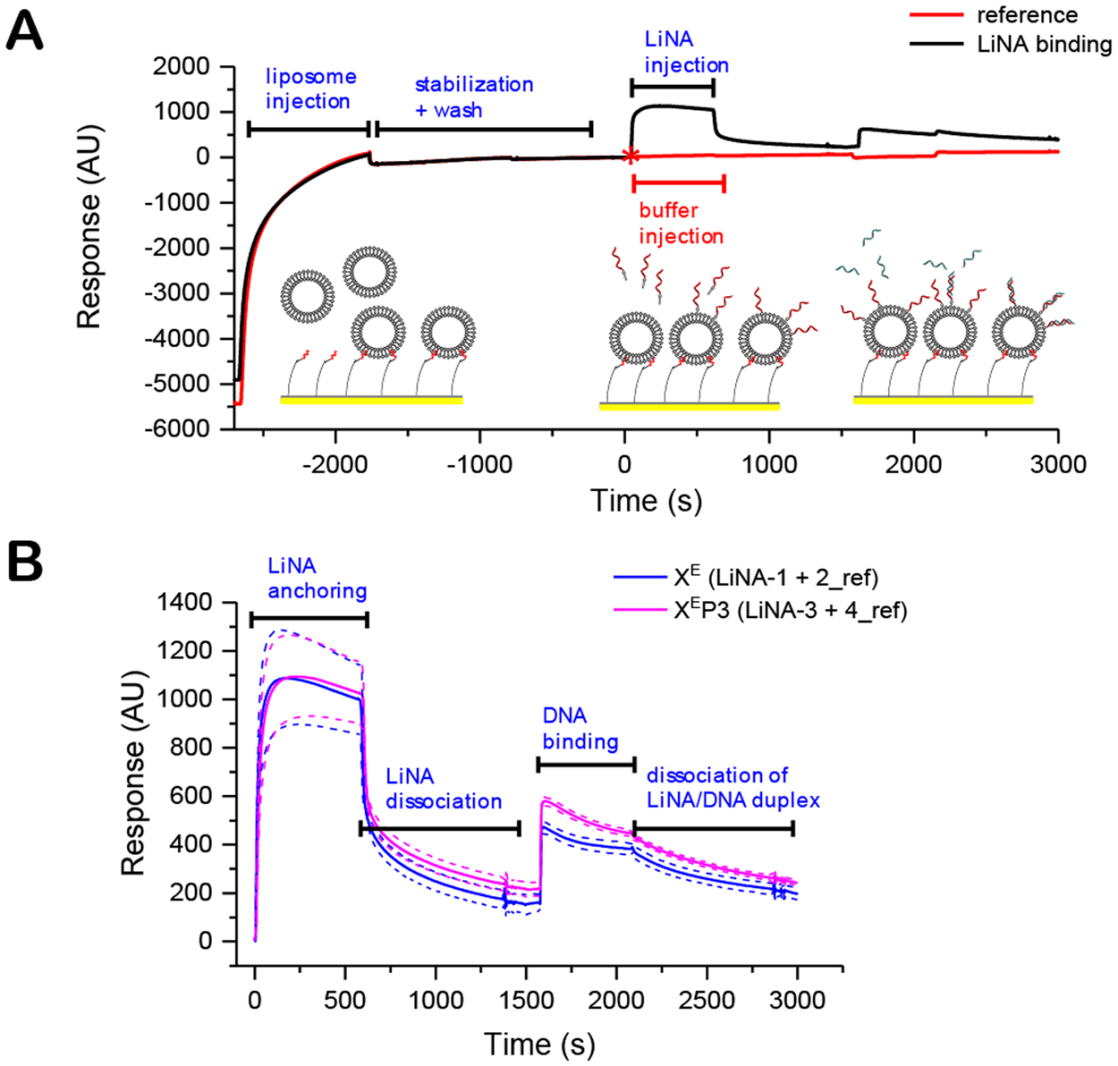
SPR detection of LiNA-binding to immobilized liposomes. (**A**) Surface Plasmon Resonance (SPR) assay for detecting spontaneous binding of LiNAs to surface immobilized liposomes. After liposome capture (2 µl/min, 900 seconds), equilibration period (900 s) and buffer wash (20 µl/min) LiNA solution (5 µM in HBS, 600 s, 2 µl/min) or HBS (reference measurement) was injected followed by a dissociation phase under a flow of HBS (600 s, 2 µl/min). Then, complementary DNA (without lipid-modification) is injected in the same manner in both experiments. The complementary oligonucleotides showed no affinity to the liposomes on the sensor surface. The response and time axes of both runs are aligned at the point of LiNA injection (red asterisk) and the reference values subtracted. (**B**) Average response of LiNA-insertion for LiNA-1 (X^E^) and LiNA-3 (X^E^P3) and subsequent hybridization of complementary DNA to LiNA strands that remain bound to the surface after the dissociation phase. Measurements carried out at 25 °C, the solid line shows the mean signal of two injections, the dotted lines show the total error.

The spontaneous anchoring of LiNAs into liposome bilayers was assayed using surface plasmon resonance, a method that generates a signal based on the bulk of material present a few hundred nanometers above a gold surface. Liposomes were immobilized onto a sensor chip coated with an alkyl-modified dextran matrix (Biacore L1 sensor chip) in the instrument flow cell (Fig. 5A)^40^. LiNAs were injected slowly (contact time 0-600 s, 2 µl/min, Fig. 5B) giving rise to a fast binding response, saturating the surface of the immobilized liposomes within 60 seconds. After the initial contact time, a continuous flow of 2 µl/min HBS resulted quick removal of loosely associated LiNA, but thereafter the signal approached a new baseline due to the remaining anchored LiNAs (+200-300 response units after 1000 s). LiNA presence was corroborated by testing their ability to form duplexes with complementary DNA strands (non-modified), which were likewise injected slowly (contact time 1600–2100 s, 2 µl/min) giving rise to approximately a doubling of the response at the end of contact time.

This is expected for an efficient hybridization as the surface bound mass approximately doubles when LiNAs hybridize with their complementary strand. After contact of the complementary DNA, a slow signal decrease was observed during HBS flow over the surface, which must either be due to de-hybridization of DNA or “de-anchoring” of LiNA/DNA duplexes (anchoring must become less effective after nearly doubling the molecular mass by a second hybridized DNA).

### LiNA-induced liposome fusion and content mixing

For fusion experiments, the fusogens LiNA1-4 were engrafted onto liposomes (DOPC/DOPE/Cholesterol, 2:1:1, ~135 nm avg. diameter (Figure S3), incubated with LiNAs for 15 min at r.t), thereby providing two populations of liposomes each carrying one of the complementary strands of a LiNA pair. The LiNAs hybridize upon mixing the two populations causing the liposomes to cross-link and fuse. Figure 4 schematically illustrates LiNA functionalized liposomes and the recognition sequences used in the present study.

**Figure 6:**
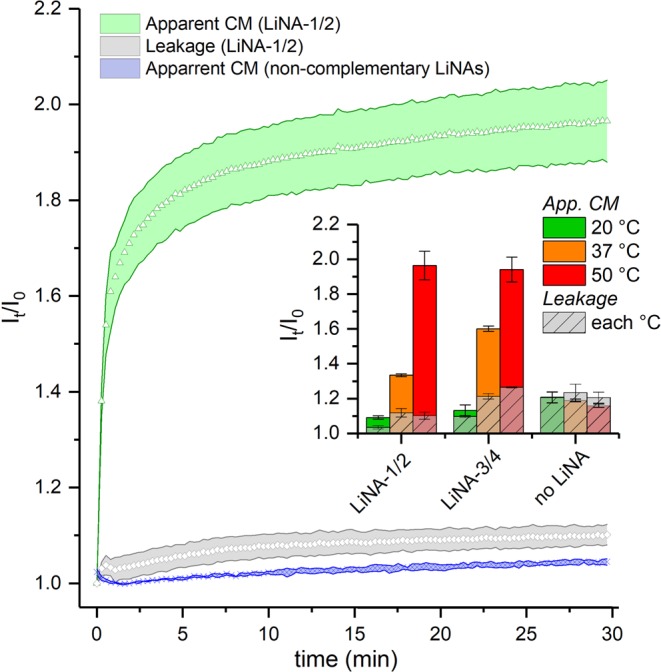
Content mixing during fusion experiments. Increase in relative fluorescence during time course experiments upon mixing populations functionalized with LiNA-1/2 (Apparent CM, green) including controls, i.e. leakage during fusion (grey) and apparent CM when LiNAs are non-complementary (blue). Obtained at 50 °C, ratio labeled/unlabeled liposomes 1:3, ~195 and 65 LiNA/liposome, respectively. The inset summarizes the observed data with complementary LiNAs, or in absence of LiNAs, illustrating contribution of leakage to the observed signal. Error bars: total error between duplicates.

The fusion efficiency was assayed by measuring the content mixing (CM) of the two liposome populations using a well-established fluorescence assay. One population encapsulates Sulforhodamine B (SRB) at a self-quenching concentration (20 mM)^21,41^ while the other is filled with buffer (unlabeled). In brief, the assay works as follows: upon opening of a fusion pore between docked liposomes, the content volumes rapidly mix and SRB is diluted causing an increase in fluorescence signal intensity (dequenching). SRB efflux and water influx (in the following termed “leakage”) also lead to signal increase. Such leakage could either occur passively or as a result of membrane lesions during fusion. Thus, we refer to this experiment as “apparent CM” (Fig. 6, green curve) and for each experiment a leakage control was performed in parallel. For these controls both populations contained SRB at the same concentration and content mixing thus gives rise to zero dilution. Any observed signal increase must, in this case, be due to SRB-dilution via leakage processes (Fig. 6, grey curve). In another control, the measurement of the apparent CM was repeated with non-complementary LiNAs (LiNA-1 + LiNA-1, Fig. 6, blue curve). All fusion experiments were carried out at 20, 37 and 50 °C and in independent duplicates. The approx. number of LiNA strands on the SRB-labeled and unlabeled liposomes were 195 and 65, respectively, and the populations were mixed in a 1:3 ratio. This allows each SRB-labeled liposome to statistically fuse with up to three unlabeled ones, diluting encapsulated [SRB] from 20 mM to 10 mM, 6.7 mM and 5 mM, after the first, second and third round, respectively.

In addition to measuring CM, the particle size distribution was measured at intervals throughout the course of the experiment. This was done by taking aliquots from a fusion experiment using only unlabeled liposomes and diluting these approx. 50-fold in buffer at room temperature. The samples were then analyzed using nanoparticle tracking analysis (NTA, see Figures S4 and S5).

As previously observed, the LiNAs mediated fusion most efficiently at 50 °C as summarized in Fig. 6. The inset illustrates the amount of leakage that contributed to the apparent CM signal, from which it is clear, that in absence of LiNAs the signal increase was due to background processes entirely (see Figure S6 for time course overlay with control experiments). At the same time, the presence of non-complementary LiNAs only gave rise to a minute signal increase (Fig. 6, blue curve) suggesting that engrafting liposomes with polyaza crown ether modified LiNAs had the effect of decreasing passive SRB permeability as observed previously for the anchors with a 3-aminopropane-backbone^12,13^. As hypothesized earlier, this effect is likely due to the charge repulsion between liposomes by the polyanionic DNA on the liposome surface^12,42^.

In the current study, elevated leakage was observed for LiNAs with a P3 spacer (LiNA-3/4), where both leakage and content mixing scaled with temperature, in comparison to LiNA-1/2, where leakage remained low. In previous LiNA systems, based on a simpler anchor structure (3-aminopropane-1,2-diol)^12^, the presence of a P3 spacer did not lead to an increased leakage. Based on this result, we speculate, that the spacer can affect membrane stability.

After correcting for leakage contribution to the apparent CM, the resulting net signal was used to estimate the percentage of full fusion events of SRB-filled liposomes relative to the total number of SRB-filled liposomes, hereafter termed *fusion yield*. To this end, a calibration curve relating the net I/I_0_ to the remaining fraction of liposomal SRB-concentration (*χ*_*SRB*_) follows:

$$I/{I}_{0}=\,a\ast ({\chi }_{SRB})+b={I}_{\chi }/{I}_{\chi =1},$$

where ${I}_{\chi }/{I}_{\chi =1}$ is the intensity of a series of samples with different *χ*_*SRB*_ (*χ*_*SRB*_ = 0.8, 0.5, 0.33; corresponding to [SRB] 16 mM, 10 mM or 6.7 mM, respectively) divided by the intensity at the initial SRB concentration ([SRB]_0_ = 20 mM); *a* and *b* are linear coefficients and were found to be independent on the temperature at which the samples were recorded (see Methods and Ref.^13^). The fusion yield was then defined as following:

$${\rm{fusion}}\,{\rm{yield}}={{\chi }_{SRB}}^{-1}\ast 100 \% .$$

**Figure 7:**
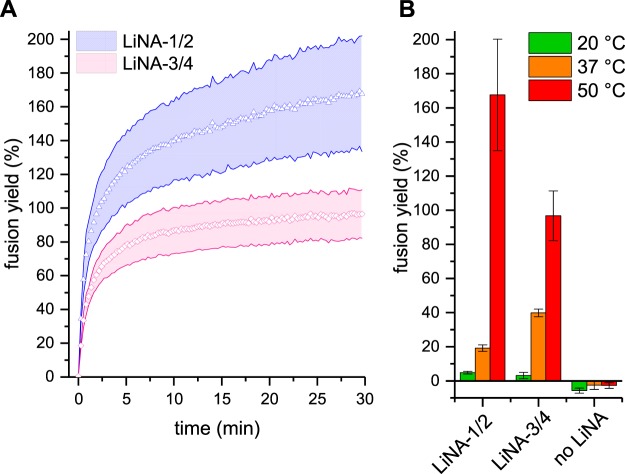
Calculated fusion yield. Fusion yield of SRB-labeled population after leakage correction and applying standard curve data of a series of liposome-encapsulated SRB (see SI). (**A**) Time course experiments depicting fusion yield over time for XE (LiNA-1/2) and XE-P3 (LiNA-3/4) systems at 50 °C; (**B**) Summary of calculated fusion yields after 30 min at the indicated temperatures. Error bands/bars represent total error between duplicate experiments.

As indicated above, yields >100% can occur, which signifies that labeled liposomes on average fused with more than one unlabeled liposome. The fusion yields at 50 °C are plotted as a function of time in Fig. 7A, and the yields at 30 min are summarized in Fig. 7B.

The temperature dependence of LiNA-induced membrane fusion; ~5% fusion yield at 20 °C, 20-40% at 37 °C and 95-170% at 50 °C (Fig. 7B); is congruent with the trend of increasing spontaneous fusion with temperature^29^. Temperature increase has a two-fold effect: i) increasing the energy of thermal collisions between membranes brought in proximity by the hybridized LiNAs and ii) decreasing the interfacial tension of the membrane due to an increased surface area – each lipid simply has a higher effective surface area^43^. The consequence of this effect was demonstrated by Parolini et al. using giant liposomes that were tethered to each other via long oligonucleotides: microscopy images showed that the contact area between the liposomes increased with temperature, without changing the number of available tethers^44^. For LiNA induced fusion, this finding suggests that higher temperatures support membrane deformation, increasing inter-liposome contact area and the number of LiNA duplexes acting cooperatively, prolonging contact time, again amplifying the number thermal collisions between liposomes. Furthermore, decreased surface tension is supportive of extreme membrane curvature, i.e., negative during fusion stalk formation and highly positive during pore opening^45,46^. A similar trend was observed by Sadek *et al*. in the membrane fusion mediated by a β-peptide nucleic acid based SNARE-mimic^47^.

In the present study, the more rigid pair LiNA-1/2 exhibited a higher maximal fusion yield (~170%) when compared to LiNA-3/4 (~95%), a result that might be explained by the shorter intermembrane distance LiNA-1/2 system, than in presence of the two P3 spacers. In contrast, the apparent CM signal was comparable between these systems, which prompts us to speculate whether the triethylene glycol linkers in the LiNA-3/4 play a role in the process from docking to fusion. If the linkers are innocent, the observed increase in leakage could be explained by a prolonged docked state in a configuration that is not quite as inducive to fusion than in the case of LiNA-1/2. If, however, the ethylene glycol interacts strongly with the lipid headgroups of the membrane, it might increase fusogenicity and permeability by disturbing the hydration layer around the membrane. We looked at the initial fusion rates (dYield/d*t*_t=0_) of the studied systems and compared them with other LiNA pairs from previous studies (see Table 1). The data suggests that the addition of a spacer slowed down the observed fusion rate both in case of the aza-crown ether and the aminopropane backbone.

**Table 1:** Comparison of fusion yield and initial rates at 50 °C.

Strands	Anchor Design	Fusion yield at 30 min (%)^b^	Initial fusion rate (%/min)^b,c^
LiNA-1/2	T-X^E^-17nt	170 ± 25	110 ± 10
LiNA-3/4	T-X^E^-P3-17nt	95 ± 10	65 ± 5
LiNA-ref1/ref2^a^	T-X^N^-17nt	105 ± 5	85 ± 5
LiNA-ref3/ref4^a^	T-X^N^-P3-17nt	280 ± 50	70 ± 5

^a^Data from ref.^13^, see SI for oligonucleotide details. ^b^Rounded to nearest 5%, ± = standard error. ^c^Linear fit to first three data points, see Figure S7.

The initial fusion rate for LiNA-1/2 was found to be very high (100% fusion yield within ~2 minutes). In the presence of the P3 linker (LiNA-3/4), the initial rate was slower. A similar trend was observed for LiNAs with the aminopropane backbone, that is, for LiNA-ref1/ref2 and LiNA-ref3/ref4, respectively (Table 1). The system with P3 and aminopropane backbone (LiNA-ref3/ref4), however, showed the highest fusion yield, i.e. the SRB-labeled population in average fused with 2.8 of 3 equivalents of unlabeled liposomes, as reported earlier, despite the lower initial rate. For the best aza-crown ether anchored setup (LiNA-1/2), the labeled liposomes fused with 1.7 equivalents of unlabeled liposomes on average. Based on these and the current results, we were intrigued to further understand the parameters controlling fusion kinetics. Different anchor designs may benefit from different optimal linker. On the other hand, linker length is inversely proportional to fusion rate. In an elegant array of coiled-coil based fusogens with different linker lengths, Daudey *et al*^39^. were able to suggest optimal linker lengths for phospholipid and cholesterol anchors, respectively. Also, for cholesterol-anchored peptides, a slightly shorter linker on one of the fusogens resulted in increased initial rate. Our previous work tested many combinations of linker positions, lengths and anchor structure based on the aminopropane design, were similar trends in fusion efficiency were observed. On the other hand, it is more challenging to rationalize the influence of backbone structure as well as the spacing between and structure of anchor-moiety on fusion efficiency. Nonetheless, the fact that structurally rather different anchor designs could both lead to very efficient fusion underlines the robustness of using LiNAs as a tool for programmable liposome fusion.

## Conclusion

Herein we describe the development of new lipid-nucleic acid conjugates (LiNAs) able to efficiently induce liposomal fusion of inner membrane leaflets and subsequently enable efficient content mixing. An improved synthesis of anchor building blocks based on a polyaza crown ether scaffold with flexible late stage introduction of lipid moieties is reported. LiNAs anchored into the outer liposomes leaflet, these anchors provided strong fusogens. When labeled liposomes were fused with 3:1 excess of unlabeled ones, rapid and efficient fusion was observed. The best system provided complete turnover of the labeled population with low leakage after only 2 min when fusion was carried out at 50 °C. While anchors used in this study are structurally rather different from our previously reported LiNAs, the high fusion efficiency at 37 and 50 °C reported in our earlier study was reproduced. The findings underline LiNA-based fusogens as a robust tool for the fusion of biological membranes.

## Methods

### Materials and general methods

1,2-Dioleoyl-*sn*-glycero-3-phosphocholine (DOPC), 1,2-dioleoyl-*sn*-glycero-3-phosphoethanolamine (DOPE), 1,2-dipalmitoyl-*sn*-glycero-3-phosphoethanolamine-*N*-(7-nitro-2-1,3-benzoxadiazol-4-yl) ammonium salt (DPPE-NBD), 1,2-dipalmitoyl-*sn*-glycero-3-phosphoethanolamine-*N*-(lissamine rhodamine B sulfonyl) ammonium salt (DPPE-LissRhod) were purchased from Avanti Polar Lipids. Cholesterol (Chol) was purchased from Riedel-de Haën in extra pure quality. 9-*O*-Dimethoxytrityl-triethylene glycol 1-[(2-cyanoethyl)-(*N*,*N*-diisopropyl)]-phosphoramidite (Spacer 9, modification P3) was purchased from Glen Research and other phosphoramidites from Proligo. All other reagents were purchased from standard suppliers in reagent grade purity or higher. High purity water (MilliQ, Merck Millipore Advantage A10 system) was used throughout. The liposome fusion experiments, thermal denaturation, circular dichroism (CD) and membrane binding experiments were all carried out in a HEPES buffered saline (HBS, 10 mM 4-(2-hydroxyethyl)-1-piperazineethanesulfonic acid (HEPES), 100 mM NaCl in water, adjusted to pH 7.0 with a NaOH solution ([Na^+^] = 108 to 110 mM). Fluorescence spectroscopy was performed on a Varian Cary Eclipse Fluorescence Spectrophotometer equipped with a 4 position multi-cell holder connected to a Varian Temperature Controller (Peltier device heater; regulated with flow of liquid coolant). Hydrodynamic diameters were determined on a Malvern Nanosight LM14 instrument (details follow below).

### Synthesis and characterization of lipid-nucleic acid conjugates

Solid-phase automated DNA synthesis was used to synthesize LiNAs, incl. the DMT-protected phosphoramidite building blocks **5a** (0.05 M in DCE/ACN, 2:1, 400 µl, 20 min reaction time) either near the 5′- or 3′-end as previously described^3^. All LiNAs were purified using RP-HPLC after final DMT-cleavage (Table S1) and verified by MALDI-TOF mass spectrometry. Non-modified sequences (Sigma-Aldrich) were used as received. LiNA hybridization was verified by thermal denaturation experiments (Abs. 260 nm (A_260_), 20-85 °C and by CD spectroscopy. LiNA stock solutions (50% acetonitrile/water) were kept at 4 °C. Prior to fusion experiments, LiNAs were diluted to 1 µM in HBS (based on A_260_ and calculated extinction coefficients, stored at RT and used within 48 h (1 µM solutions stored at 4 °C showed surface adsorption issues).

### General liposome preparation

To produce unlabeled (DOPC/DOPE/Chol; 2:1:1, molar ratio) or membrane-labeled (additionally 0.25% Lissamine-Rhodamine-DPPE) lipid films, stock solutions in chloroform or methanol were mixed and evaporated under a stream of N_2_ and further dried under high vacuum. The lipid films were rehydrated in buffer or SRB solution (below). The suspension was vortexed and extruded 21x through a 100 nm polycarbonate membrane (Whatman Nucleopore Track-Etched Membranes) using a hand-extruder (Avanti Polar Lipids) to form unilamellar liposomes (stored at room temperature and used within 36 h. The mean diameter was determined to be 134 ± 3 nm by Nanoparticle Tracking Analysis (NTA).

### Encapsulation of Sulforhodamine B (SRB)

Liposomes for content mixing and leakage experiments were obtained by rehydrating lipid films HBS containing 20 mM SRB, placed in a bath sonicator for 10 min at 50 °C, extruded as above and used within 36 h (mean particle diameter 135 ± 4 nm). To remove unentrapped dye, 50 µL batches of the suspensions were purified by spin-column size exclusion chromatography (GE Healthcare Illustra Microspin S-200 HR Columns) pre-equilibrated with HBS. Relative concentrations after spin-column purification was determined by fluorimetry using liposomes labeled with 0.25 mol% DPPE-Lissamine Rhodamine. After size exclusion, the following aliquots were mixed and filled to 100 µl with HBS: *blank* (10 µL Triton X-100 (1 wt%)), *reference* (10 µL of untreated liposome dispersion, 10 µL Triton X-100 (1 wt%)) and *sample* (10 µL of spin-column eluate, 10 µL Triton X-100 (1 wt%)). Average fluorescence was measured from five replicates (excitation wavelength (λ_ex_), 560 nm; emission wavelength (λ_em_), 583 nm; emission (em.) filter open, excitation/emission (ex./em). slits 5 nm, PMT 550V) and concentration determined from the average intensity (I) between three independent preparations using the formula: c = (I_*sample*_ − I_blank_)/(I_*reference*_ − I_*blank*_) × c_stock._ Measurement in presence of detergent Triton X-100 (i.e. measuring a non-liposomal dispersion) gave the best reproducibility when using 384-well plates).

### Sulforhodamine B (SRB) content mixing assay

Upon content mixing (CM) with unlabeled liposomes – or leakage into the outer medium – the dye is diluted, leading to an increase in fluorescence. The measured fluorescence increase is thus based on both content mixing and leakage (Apparent CM) was corrected by a leakage (L) control, where both liposome populations contain the SRB and fluorescence increase must stem from leakage.

#### Apparent CM (complementary and non-complementary)

SRB-filled liposomes ({SRB}) and unlabeled liposomes ({buffer}) were prepared by incubation with the respective LiNA for 15 min. at room temperature. Samples were equilibrated to the experiment temperature prior to mixing ({SRB}/{buffer} = 1:3)) in a quartz cuvette (500 µl). SRB fluorescence (λ_ex_, 545 nm; λ_em_, 583 nm, em. filter 550-1100 nm, ex./em. slits 5 nm, PMT 550V) was monitored for 30 min after population mixing at controlled temperature (cell block as temperature reference, cuvettes pre-heated for ≥ 5 min). Final concentrations: {SRB}, 46 µM total lipid concentration ([lipid] = [DOPC] + [DOPE] + [Chol]), 0.105 µM DNA (encapsulating 20 mM SRB, lipid/DNA ratio 437:1, ~195 strands per 100 nm liposome); {buffer} = 0.138 mM [lipid], 0.105 µM DNA, lipid/DNA ratio 1310:1, ~65 strands per 100 nm liposome).

#### Leakage

Two populations of SRB-filled liposomes were incubated with complementary LiNAs, equilibrated, mixed and monitored as above. Final concentrations: {SRB}^I^ and {SRB}^I﻿I^ both 46 µM [lipid], 0.105 µM DNA (encapsulating 20 mM SRB, lipid/DNA ratio 437:1, ~200 strands per 100 nm liposome)

### Calibration of SRB assay

A calibration curve with standard samples with different fractions of the starting concentration of entrapped SRB (*χ*_*SRB*_) were measured. When normalizing the signal to the intensity measured at the starting point for content mixing (*i*.*e*., I/I_0_ = Iχ/Iχ_=1_) a linear relationship to *χ*_*SRB*_ was obtained. The normalized signal was largely independent on temperature (20, 37, 50 °C) and current liposome concentration (80, 138 and 275 µM total lipid concentration). The giving the effective χ_SRB_ was calculated for each sample based on the SRB concentration after lysis in 0.1% w/v Triton X-100 (obtained against the standard curve of unentrapped SRB). The linear regression in the main text was calculated from a concatenate plot of three samples for each *χ*_*SRB*_ = 1, 0.8, 0.5, 0.33; corresponding to [SRB] 20 mM,16 mM, 10 mM or 6.7 mM, respectively.

$$I/{I}_{0}={I}_{\chi }/{I}_{\chi =1}a\ast ({\chi }_{SRB})+b=1.39\times {\chi }_{SRB}+2.38,$$

### Calculation of effective content mixing

Data from fusion experiments was expressed as I/I_0_, i.e., the fluorescence intensity at time t (I_t_) divided by the initial intensity (I_0_). Then, the I_t_/I_0_ values for leakage (L) signal were subtracted from I_t_/I_0_ for apparent CM giving the net. fluorescence increase (CM - L). The calibrated content mixing was calculated as χ_SRB_ = *(2*.*38 - I*_*t*_*/I*_*0*_*)/1*.*39* using the above linear relation. The fusion yield was calculated as described in the text.

### Nanoparticle Tracking Analysis (NTA) *Size distributions*

The size distributions of freshly extruded liposomes (DOPC, DOPE, Cholesterol (Chol); 2:1:1, molar ratio) and samples from fusion experiments were analyzed using a NanoSight (Wiltshire, UK) instrument, equipped with the NanoSight LM14 flow-cell and laser assembly (405 nm diode laser), a 20 × objective and CCD camera. Data was recorded and analyzed using the NTA v. 2.3 software (Recording: 5 × 30 s with Shutter 387 (25 fps), Gain 200-350, Histogram: 520 lower threshold); Analysis: Detection threshold 10, Blur ‘auto’, Min. Track Length 10, Min. expected size ‘auto’). Liposome preparations were measured at 2 or 5 µM total lipid concentration.

#### NTA-monitored fusion

Samples were prepared identically as for content mixing. Plain liposomes were used for both populations a 1:1 ratio (I, 138 µM [lipid] with 0.105 µM LiNA-1 and II, 138 µM [lipid] with 0.105 µM LiNA-2, lipid/DNA ratio 1310:1, 65 strands per 100 nm liposome, 15 min incubation at room temperature). The two different liposome populations were pre-heated to 50 °C, then mixed and incubated for to 30 min at 50 °C. At 1, 5, 10, 15 and 30 min, aliquots of the fusion mixtures were transferred and diluted [lipid] = 5 µM (55-fold dilution) in buffer at 20 °C and analyzed as immediate as possible. The strong dilution effectively slowed down further fusion and aggregation processes. The t_0_ sample corresponds a preparation with unpaired oligonucleotide (LiNA-1) after 15 min incubation. A non-complementary control (LiNA-1/1) was prepared and analyzed after 30 min at 50 °C.

### Surface Plasmon Resonance (SPR) Binding Assay

SPR sensorgrams were recorded on a Biacore^TM^ X100 system using L1 sensor chip (GE Lifesciences) at 25 °C. A method for immobilizing lipidated DNA, introduced by Makishi *et al*., was used with modified conditioning^40^. The conditioning of the L1 chip was done by four injections of iPrOH/50 mM NaOH 4:6 (120 s, flow rate: 10 µL/min). After the injection and immobilization of plain liposomes (DOPC, DOPE, Chol, 2:1:1, molar ratio, nominal diameter 100 nm, 1 mM, 1200 s, flow rate: 2 µL/min). Excess liposome material was removed by increasing the flow rate of the running buffer to 10 µL/min for 300 s to achieve a stable baseline (treatment with dilute alkaline was avoided due to the presence of ionizable DOPE). The lipidated DNA was injected (5 µM either LiNA-1 or LiNA-3, or running buffer (negative control); 600 s, flow rate: 2 µL/min), followed by a flow of running buffer to enable dissociation (800 s, flow rate: 2 µL/min). Subsequently, an unmodified complementary or non-complementary DNA strand was injected (A’, 5 µM, 600 s, flow rate: 2 µL/min) After a dissociation time (800 s, flow rate: 2 µL/min), the L1 chip was regenerated by two injections of 50 mM NaOH in 50% v/v isopropanol/water (20 µL, flow rate: 10 µL/min).

## Supplementary information

Lipidated Polyaza Crown Ethers as Membrane Anchors for DNA-Controlled Content Mixing between Liposomes
