# Supplementary material for: Lipidated Polyaza Crown Ethers as Membrane Anchors for DNA-Controlled Content Mixing between Liposomes
Source: Sci Rep. 2019 Sep 25;9:13856. doi: 10.1038/s41598-019-49862-y (PMC6761097; doi:10.1038/s41598-019-49862-y)
Supplement: Supplementary file 1 — Lipidated Polyaza Crown Ethers as Membrane Anchors for DNA-Controlled Content Mixing between Liposomes [file 41598_2019_49862_MOESM1_ESM.docx]

Supplementary Information

Lipidated Polyaza Crown Ethers as Membrane Anchors for DNA-Controlled Content Mixing between Liposomes

Philipp M. G. Löffler^1^, Anders Højgaard Hansen^1^, Oliver Ries^1^, Ulla Jakobsen^1,2^, Alexander Rabe^1^, Kristian T. Sørensen^1^, Kasper Glud^1^ and Stefan Vogel*

^1^Department of Physics, Chemistry and Pharmacy, University of Southern Denmark, Campusvej 55, DK-5230 Odense M, Denmark University of Southern Denmark.

^2^PET & Cyclotron Unit, Department of Nuclear Medicine, Odense University Hospital, Sdr. Boulevard 29, 5000 Odense C, Denmark

[*snv@sdu.dk](mailto:*snv@sdu.dk)

Table of Contents

[Oligonucleotide purification and analysis 3](#_Toc5787787)

[Synthetic procedures 3](#_Toc5787788)

[3-{bis-[2-(2-aminoethoxy)ethyl]amino}propan-1-ol (2) 3 3](#_Toc5787789)

[3-(19-{2-[bis(4-methoxyphenyl)phenyl)methoxy]ethoxy}-6,12-dioxa-3,9,15,21-tetraazabicyclo[15.3.1]henicosa-1(20),17(21),18-trien-9-yl]propan-1-ol (4) 3](#_Toc5787790)

[3-[19-{2-[bis(4-methoxyphenyl)(phenyl)methoxy]ethoxy}-3,15-dihexadecyl-6,12-dioxa-3,9,15,21-tetraazabicyclo[15.3.1]henicosa-1(21),17,19-trien-9-yl]propan-1-ol (5) 4](#_Toc5787791)

[3-[19-{2-[bis(4-methoxyphenyl)(phenyl)methoxy]ethoxy}-3,15-dihexadecyl-6,12-dioxa-3,9,15,21-tetraazabicyclo[15.3.1]henicosa-1(21),17,19-trien-9-yl]propan-1-ol (5a) 4](#_Toc5787792)

[**1H, 13C, 31P-NMR spectra of compounds 2-5a** 5](#_Toc5787793)

[^1^H-NMR spectrum of 2 (300 MHz, CDCl3, 25 °C) 5](#_Toc5787794)

[^13^C-NMR spectrum of 2 (75 MHz, CDCl_3_, 25 °C) 5](#_Toc5787795)

[^1^H-NMR spectrum of 4 (300 MHz, CDCl_3_, 25 °C) 5](#_Toc5787796)

[^13^C-NMR spectrum of 4 (75 MHz, CDCl_3_, 25 °C) 6](#_Toc5787797)

[^1^H-NMR spectrum of 5 (400 MHz, CDCl_3_, 25 °C) 6](#_Toc5787798)

[13C-NMR spectrum of 5 (101 MHz, CDCl_3_, 25 °C) 7](#_Toc5787799)

[Supplemental data and discussion 8](#_Toc5787800)

[Thermal denaturation data 8](#_Toc5787801)

[CD spectra 8](#_Toc5787802)

[Nanoparticle tracking data 9](#_Toc5787803)

[Additional data for content mixing assay 10](#_Toc5787804)

[Initial fusion rate data 11](#_Toc5787805)

Oligonucleotide purification and analysis

**Table S1.** Oligonucleotide sequences and data

| **Entry** | **Type** | **Sequence** (5' - 3') | **R_t_** [min] | **Mass (calcd.)**  [g/mol] | **Mass (found)** [m/z] | **ε_260nm_^[a]^**  [mM ^-1^cm^-1^] |
| --- | --- | --- | --- | --- | --- | --- |
| **LiNA-1** | X^E^ | T**X^E^** TGT GGA AGA AGT TGG TG | 11.9^[b]^ | 6572.1 | 6575.2 | 182.4 |
| **LiNA-2** |  | CAC CAA CTT CTT CCA CA **X^E^**T | 11.7^[b]^ | 6261.0 | 6264.9 | 160.1 |
| **LiNA-3** | X^E^P3 | T**X^E^** P3 TGT GGA AGA AGT TGG TG | 12.7^[b]^ | 6784.3 | 6785.2 | 182.4 |
| **LiNA-4** |  | CAC CAA CTT CTT CCA CA P3 **X^E^**T | 12.1^[b]^ | 6473.0 | 6171.0 | 160.1 |
| **LiNA-ref1** | X^N^ | T**X^N^** TGT GGA AGA AGT TGG TG | 10.1^[c]^ | 6265.7 | 6267.3 | 182.4 |
| **LiNA-ref2** |  | CAC CAA CTT CTT CCA CA **X^N^**T | 10.5^[c]^ | 6264.9 | 6267.6 | 160.1 |
| **LiNA-ref3** | X^N^P3 | T**X^N^** P3 TGT GGA AGA AGT TGG TG | 8.7^[d]^ | 6476.9 | 6478.7 | 182.4 |
| **LiNA-ref4** |  | CAC CAA CTT CTT CCA CA P3 **X^N^**T | 8.8^[d]^ | 6165.8 | 6171.0 | 160.1 |
| ***A*** | Ref. | TGT GGA AGA AGT TGG TG | n.a.^[e]^ | 5345.5 | verified by the supplier ^[e]^ | 174.3 |
| ***A'*** |  | CAC CAA CTT CTT CCA CA |  | 5034.3 |  | 153.2 |

[a] As calculated according to nearest-neighbour model.^1^ [b] UHPLC Gradient, RP C8, 2 ml/min, eluents: A, 0.05M triethylamine acetate, pH 7.0; B, 75% ACN in A); gradient (R_t_ [min], A:B): 0→3, 80:20; 3→18, 80:20→ 0:100; 18→26, 0:100; [c] RP C18 eluents as before, 1 mL/min; gradient (R_t_ [min], A:B): 0→1, 90:10; 1→10, 90:10→ 0:100; 10→13, 0:100; [d] RP C8, eluents as before, 2 mL/min; (R_t_ [min], A:B): 0→1, 90:10; 1→7.5, 90:10 →0:100; 7.5→15, 0:100. [e] Cartridge purified and mass verified by supplier, Sigma-Aldrich.

Synthetic procedures

**General**. 400 MHz-^1^H, 101 MHz-^13^C, and 126 MHZ-^31^P NMR spectra were recorded on a Bruker Avance III spectrometer. All ^13^C and ^31^P spectra are ^1^H-decoupled. All spectra were recorded at 25°C and were referenced internally to solvent reference frequencies. Chemical shifts (δ) are quoted in ppm, and coupling constants (*J*) are reported in Hz. Index *a* and *b* indicate diastereotopic protons. Assignment of signals was carried out using ^1^H,^1^H-COSY, HSQC and HMBC spectra obtained on the spectrometer mentioned above. ESI mass spectrometry was performed on a Bruker microTOF-Q II system.

3-{bis-[2-(2-aminoethoxy)ethyl]amino}propan-1-ol (2) 3-{Bis-[2-(2-benzylamino-ethoxy)ethyl]amino}propan-1-ol (**1**) (7.26 g, 16.90 mmol, synthesized according to ref. ^2^) was dissolved in MeOH (100 mL) and Pd(OH)_2_/C (1.82 g) was added. The solution was treated with hydrogen gas for 30 min. and stirred under a hydrogen atmosphere overnight. This procedure was repeated five times until complete conversion of the starting material was observed on TLC (treated with a 5% ethanolic solution of ninhydrine followed by heating for detection of primary amine). The catalyst was removed by filtration over Celite and washed with MeOH. The filtrate was evaporated to dryness, affording diamine **2** (3.79 g, 90%) as a brown oil.

^1^H NMR (CDCl_3_, 300 MHz): δ = 1.70 (m, 2H; CH_2_-2^B^), 2.70-2.77 (m, 6H), 2.88 (t, 4H) (CH_2_-1,8^A^, CH_2_-3^A^), CH_2_-4,5^A^), 3.15 (br s, 5H; NH_2_, OH), 3.49-3.61 (m, 8H; CH_2_-2,7^A^, CH_2_-3,6^A^), 3.76 (m, 2H; CH_2_-1^B^) ppm.

^13^C NMR (CDCl_3_, 75.5 MHz): δ = 28.52 (CH_2_-2^B^), 41.57 (CH_2_-4,5^A^), 54.20 (CH_2_-1,8^A^), 55.08 (CH_2_-3^B^), 63.57 (CH_2_-1^B^), 68.77, 72.59 (CH_2_-2,7^A^, CH_2_-3,6^A^) ppm.

MS (ESI): *m/z* calcd for C_11_H_27_N_3_O_3_ [M+H]^+^ 250.2125, [M+Na] 272.1945, found [M+H]^+^ 250.2192, [M+Na]^+^ 272.2113.

3-(19-{2-[bis(4-methoxyphenyl)phenyl)methoxy]ethoxy}-6,12-dioxa-3,9,15,21-tetraazabicyclo[15.3.1]henicosa-1(20),17(21),18-trien-9-yl]propan-1-ol (4) Dialdehyde **3** (1.99 g, 4.00 mmol) was dissolved in anhydrous MeOH (100 mL) and molecular sieves (3Å, 2.0 g) were added. A solution of diamine **2** (1.00 g, 4.01 mmol, synthesized according to ref. ^2^) in anhydrous MeOH (40 mL) was slowly added and the reaction mixture was stirred at RT for 1 h followed by reflux for 3 h. After cooling to 5 ^o^ C (ice bath) NaBH_4_ (1.22 g, 32.26 mmol) was added and the mixture was stirred for 3 days. The reaction mixture was filtered, and the solvent removed under reduced pressure. Purification by column chromatography using CHCl_3_/MeOH/NH_3_ (24% aq.) 10:1:0.1 as eluent afforded compound **4** as an orange oil (1.68 g, 59%).

^1^H NMR (CDCl_3_, 300 MHz): δ = 1.69 (m, 2H; CH_2_-2^E^), 2.67-2.75 (m, 6H), 2.81 (t, 4H) (CH_2_-1,10^A^, CH_2_-4,7^A^, CH_2_-3^E^), 3.43 (t, 2H; CH_2_-2^G^), 3.61 (m, 8H; CH_2_-2,9^A^, CH_2_-3,8^A^), 3.71-3.85 (m, 15H; CH_2_-7^B,C^, CH_2_-5,6^A^, CH_2_-1^E^, OH, NH), 4.14 (t, 2H; CH_2_-1^G^), 6.62 (s, 2H; CH-2,5^F^), 6.81-6.85 (m, 4H; CH-2,6^B,C^), 7.26-7.36 (m, 7H), 7.46 (d, 2H) (CH-3,4,5,6,7^D^, CH-3,5^B,C^) ppm.

^13^C NMR (CDCl_3_, 75.5 MHz) : δ = 28.50 (CH_2_-2^E^), 48.83 (CH_2_-4,7^A^), 54.24 (CH_2_-5,6^A^), 54.69 (CH_3_-3^E^), 54.84 (CH_2_-1,10^A^) 55.33 (CH_3_-7^B,C^), 62.17 (CH_2_-1^E^), 63.98 (CH_2_-1^G^), 67.48 (CH_2_-2^G^), 68.77, 70.48 (CH_2_-2,9^A^, CH_2_-3,8^A^), 86.34 (C-1^D^), 107.52 (CH-2,5^F^), 113.24 (CH-3,5^B,C^), 126.92 (CH-5^D^), 127.94, 128.27 (CH-3,7^D^, CH-4,6^D^), 130.17 (CH-2,6^B,C^), 136.08 (C-1^B,C^), 144.87 (C-2^D^), 158.61 (C-4^B,C^), 160.07 (C-3,4^F^), 165.89 (C-1^F^) ppm.

MS (ESI): *m/z* calcd for C_41_H_54_N_4_O_7_ [M+H]^+^ 715.4065, [M+Na]^+^ 737.3885, found [M+H]^+^ 715.4083, [M+Na]^+^ 737.4100.

3-[19-{2-[bis(4-methoxyphenyl)(phenyl)methoxy]ethoxy}-3,15-dihexadecyl-6,12-dioxa-3,9,15,21-tetraazabicyclo[15.3.1]henicosa-1(21),17,19-trien-9-yl]propan-1-ol (5) Sodium triacetoxy borohydride (594 mg, 2.80 mmol) and molecular sieves (3Å, 0.75 g) were added to a solution of compound 3.15 (500 mg, 0.70 mmol) in anhydrous DCE (5 mL) and cooled to 5 °C. Hexadecanal (0.81 mL, 2.80 mmol) in anhydrous DCE (2 mL) was added and the reaction mixture was stirred overnight, allowing it to warm to room temperature. After dilution with CHCl3, the reaction mixture was filtered, and the solvent removed in vacuo. Purification by column chromatography using CHCl_3_:MeOH:NEt_3_ 100:10:1 as eluent afforded compound 5 (477 mg, 50%, contains <1 eq. NEt3) as an orange oil.

^1^H NMR (CDCl_3_, 300 MHz): δ = 0.88 (t, 6H, CH_3_-16^H,J^), 1.17-1.31 (m, 52H, CH_2_-(3-15)^H,J^, 1.52 (m, 4H, CH_2_-2^H,J^, 1.62 (m, 2H; CH_2_-2^E^), 2.58-2.64 (m, 8H), 2.71 (t, 4H) (CH_2_-1^H,J^, CH_2_-1,10^A^, CH_2_-4,7^A^, CH_2_-3^E^), 3.36-3.48 (m, 10H; CH_2_-2^G^, CH_2_-2,9^A^, CH_2_-3,8^A^), 3.48-3.84 (m, 13H; CH_2_-7^B,C^, CH_2_-5,6^A^, CH_2_-1^E^, OH), 4.18 (t, 2H; CH_2_-1^G^), 6.81-6.89 (m, 6H; CH-2,5^F^, CH-2,6^B,C^), 7.19-7.46 (m, 9H), (CH-3,4,5,6,7^D^, CH-3,5^B,C^) ppm.

^13^C NMR (CDCl_3_, 75.5 MHz) δ = 10.14 (CH_3_-16^H,J^) 14.24, 18.58, 22.82, 27.68, 29.49, 29.83, 32.06 (CH_2_-2^E^, CH_2_-(3-15)^H,J^), 45.83 (CH_2_-4,7^A^), 53.23 (CH_2_-5,6^A^), 54.36 (CH_2_-3^E^), 54.34 (CH_2_-1,10^A^) 55.35 (CH_3_-7^B,C^) 58.58 (CH_2_-1^E^), 61.04 (CH_2_-1^G^), 62.31 (CH_2_-2^G^), 67.39, 68.77 (CH_2_-2,9^A^, CH_2_-3,8^A^), 86.34 (C-1^D^), 108.19 (CH-2,5^F^), 113.27 (CH-3,5^B,C^), 126.93 (CH-5^D^), 127.96, 128.37 (CH-3,7^D^, CH-4,6^D^), 130.21 (CH-2,6^B,C^), 136.21 (C-1^B,C^), 144.95 (C-2^D^), 158.66 (C-4^B,C^), 158.66 (C-3,4^F^), 166.14 (C-1^F^) ppm.

MS (ESI): *m/z* calcd for C_73_H_118_N_4_O_7_ [M+Na]+: 1185.8893; found 1185.8872. [M+H]^+^

3-[19-{2-[bis(4-methoxyphenyl)(phenyl)methoxy]ethoxy}-3,15-dihexadecyl-6,12-dioxa-3,9,15,21-tetraazabicyclo[15.3.1]henicosa-1(21),17,19-trien-9-yl]propan-1-ol (5a) Phosphitylation of **5** was carried out as described in Rohr et al.^2^ The product was dissolved in 2:1 DCE/ACN (dry, 3Å sieves) at 0.05 M concentration, stored in a desiccator and used within one month.

**1H, 13C, 31P-NMR spectra of compounds 2-5a**

^1^H-NMR spectrum of 2 (300 MHz, CDCl3, 25 °C)


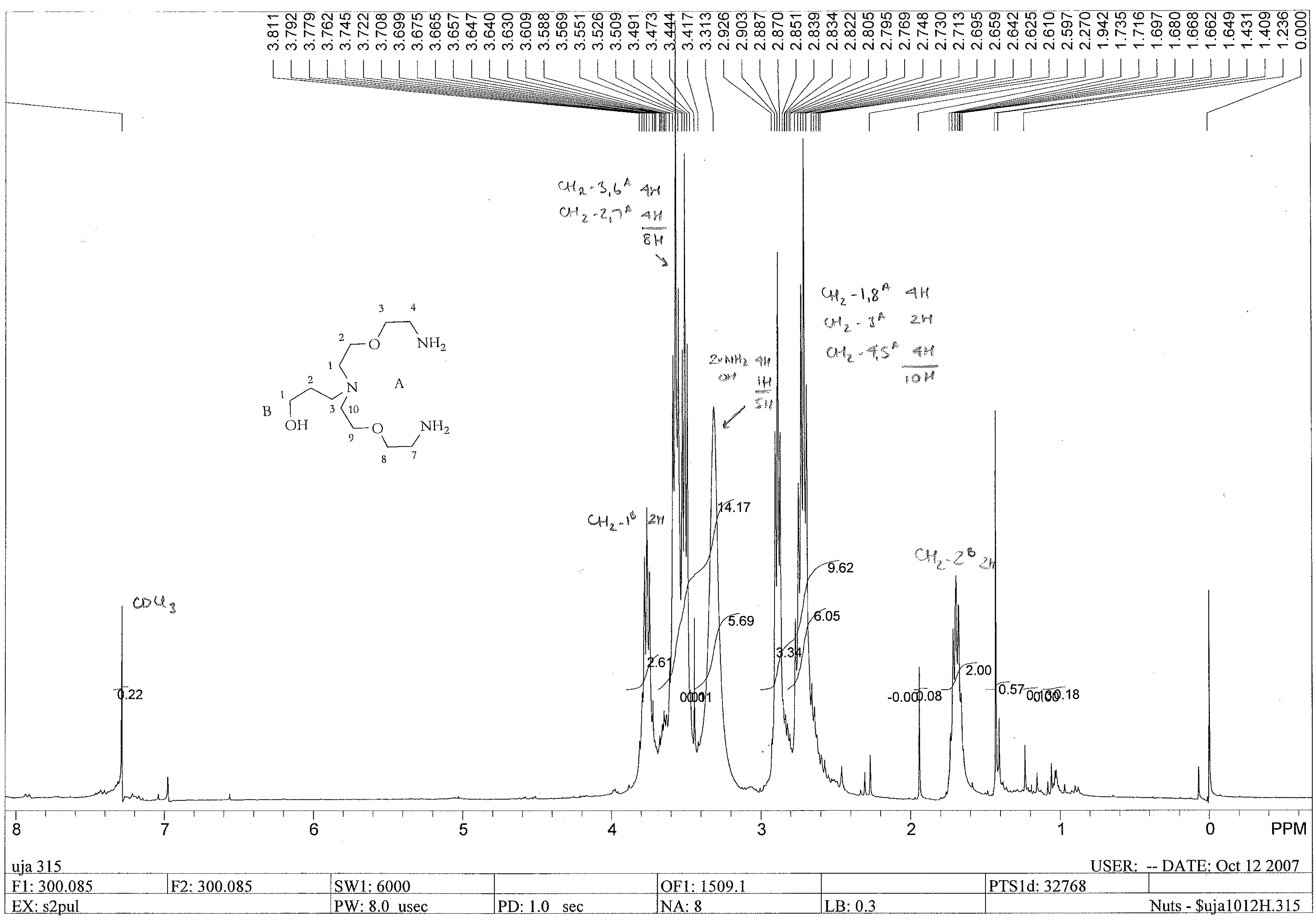


^13^C-NMR spectrum of 2 (75 MHz, CDCl_3_, 25 °C)


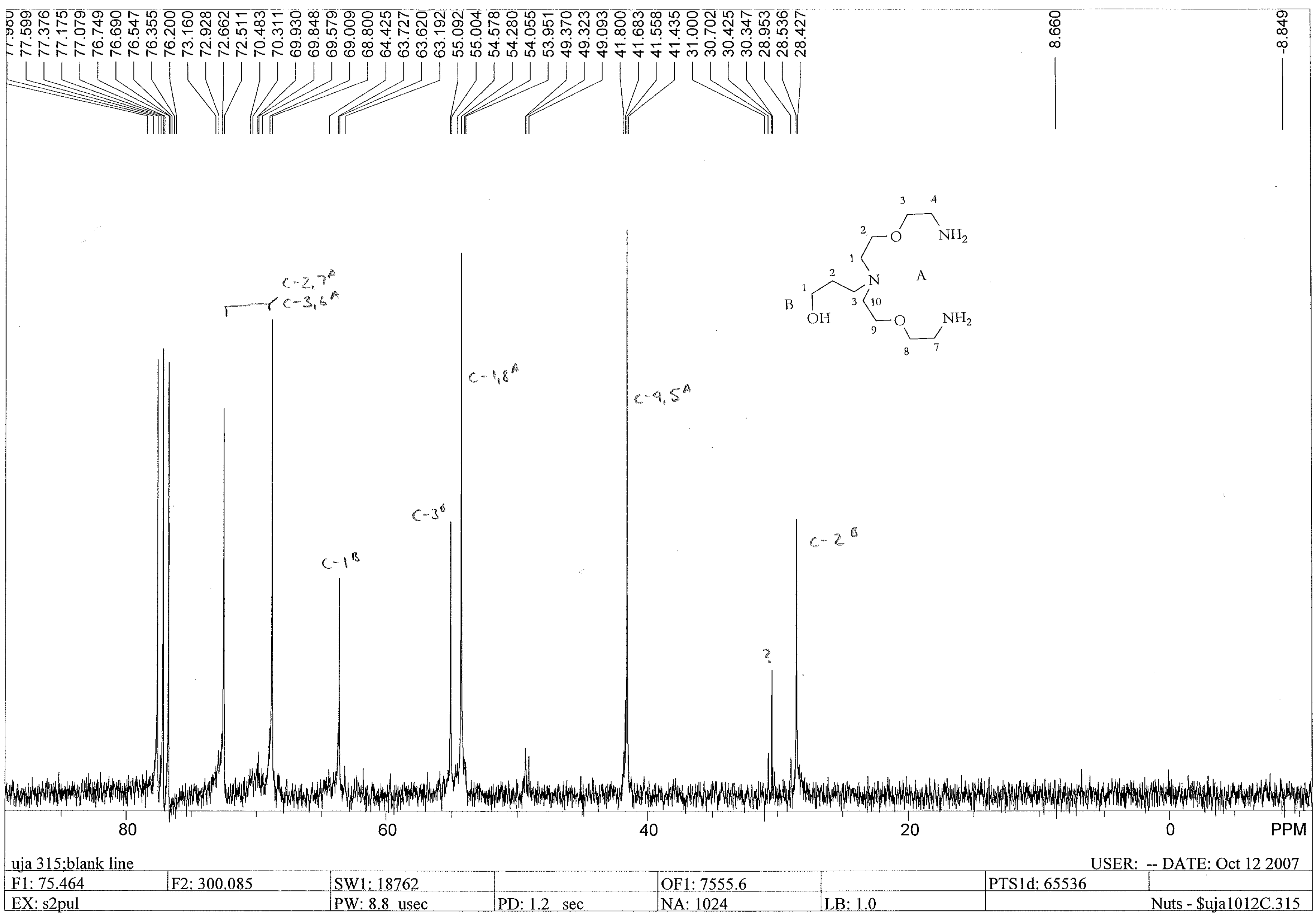


^1^H-NMR spectrum of 4 (300 MHz, CDCl_3_, 25 °C)


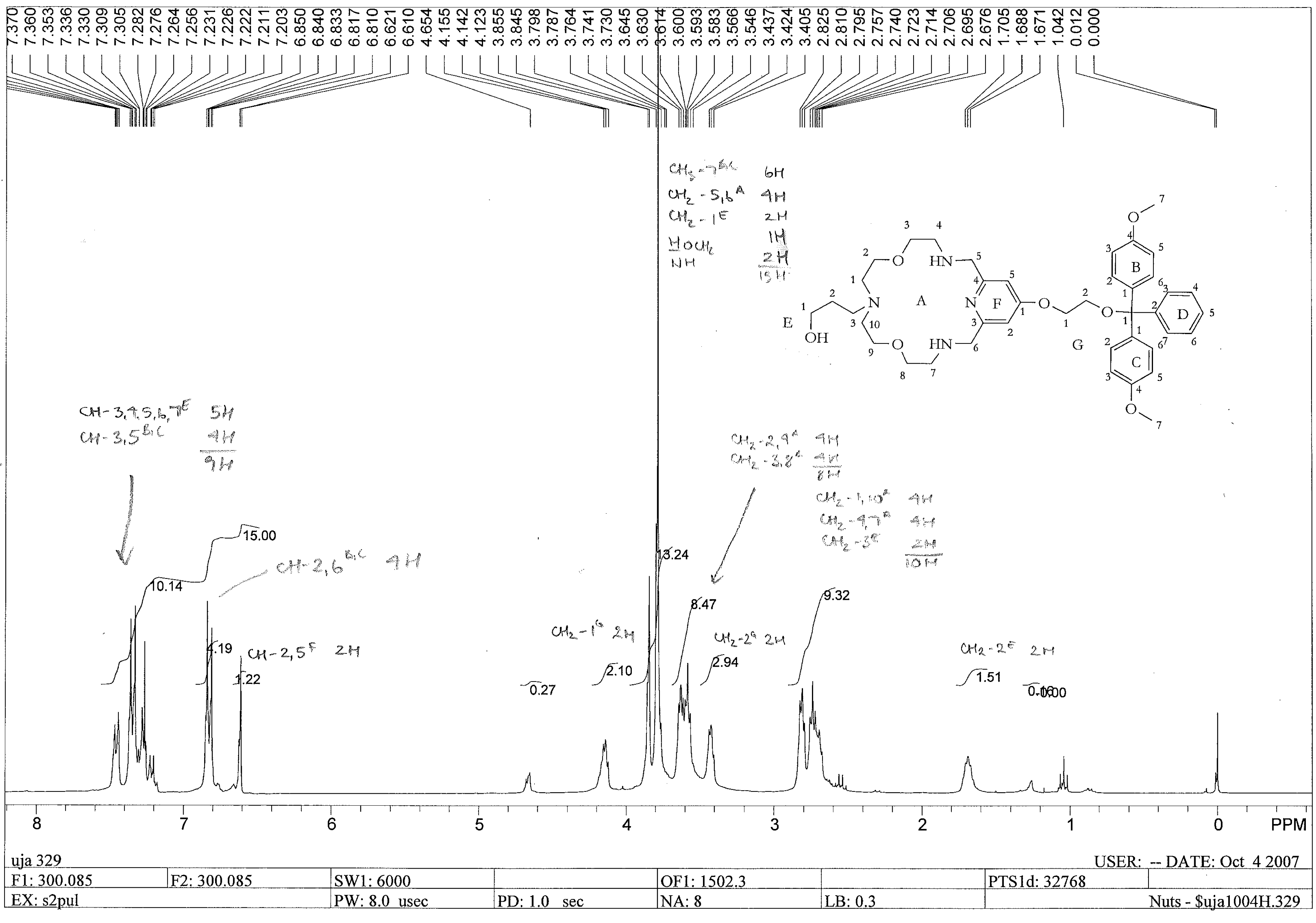


^13^C-NMR spectrum of 4 (75 MHz, CDCl_3_, 25 °C)


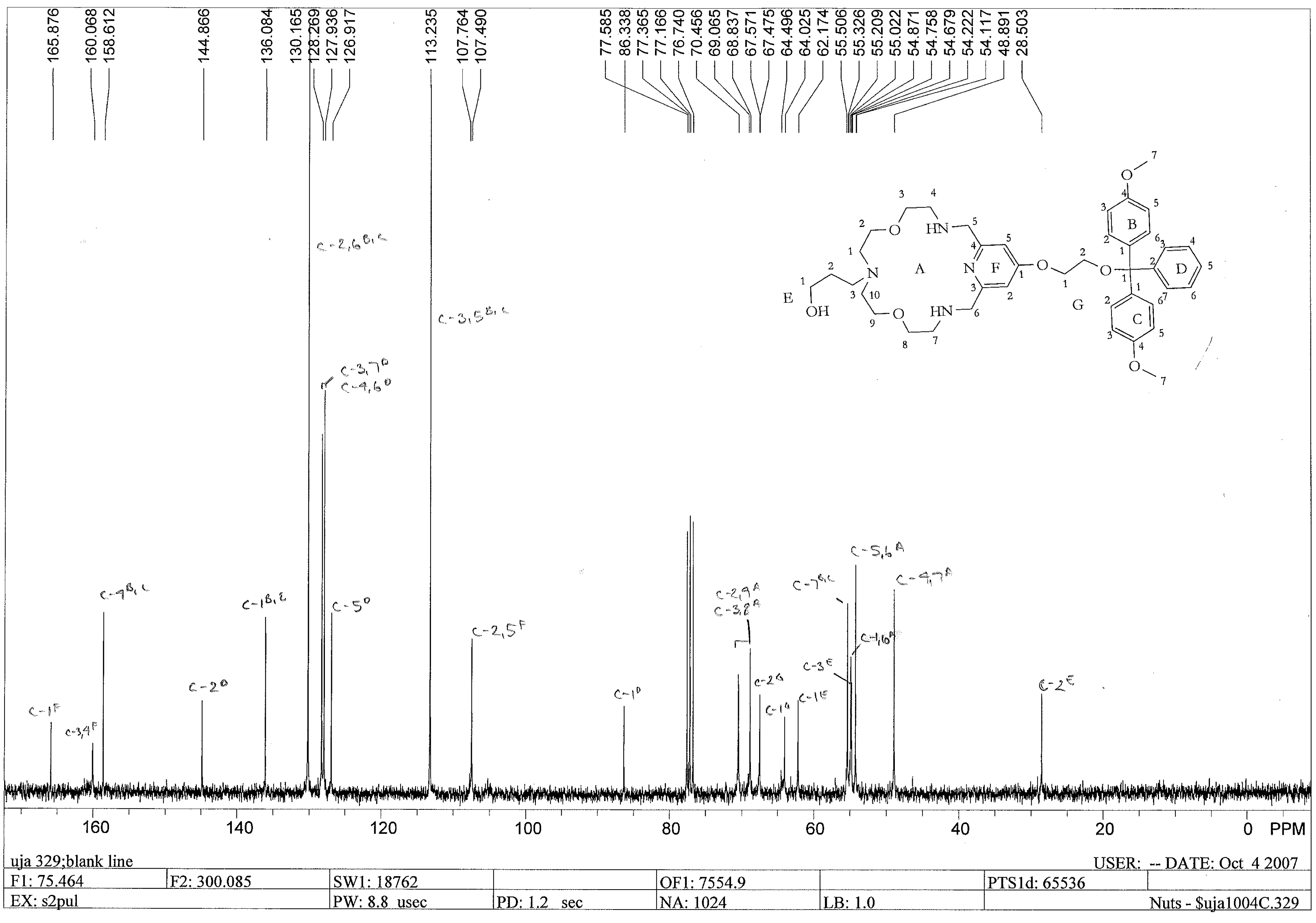


^1^H-NMR spectrum of 5 (400 MHz, CDCl_3_, 25 °C)


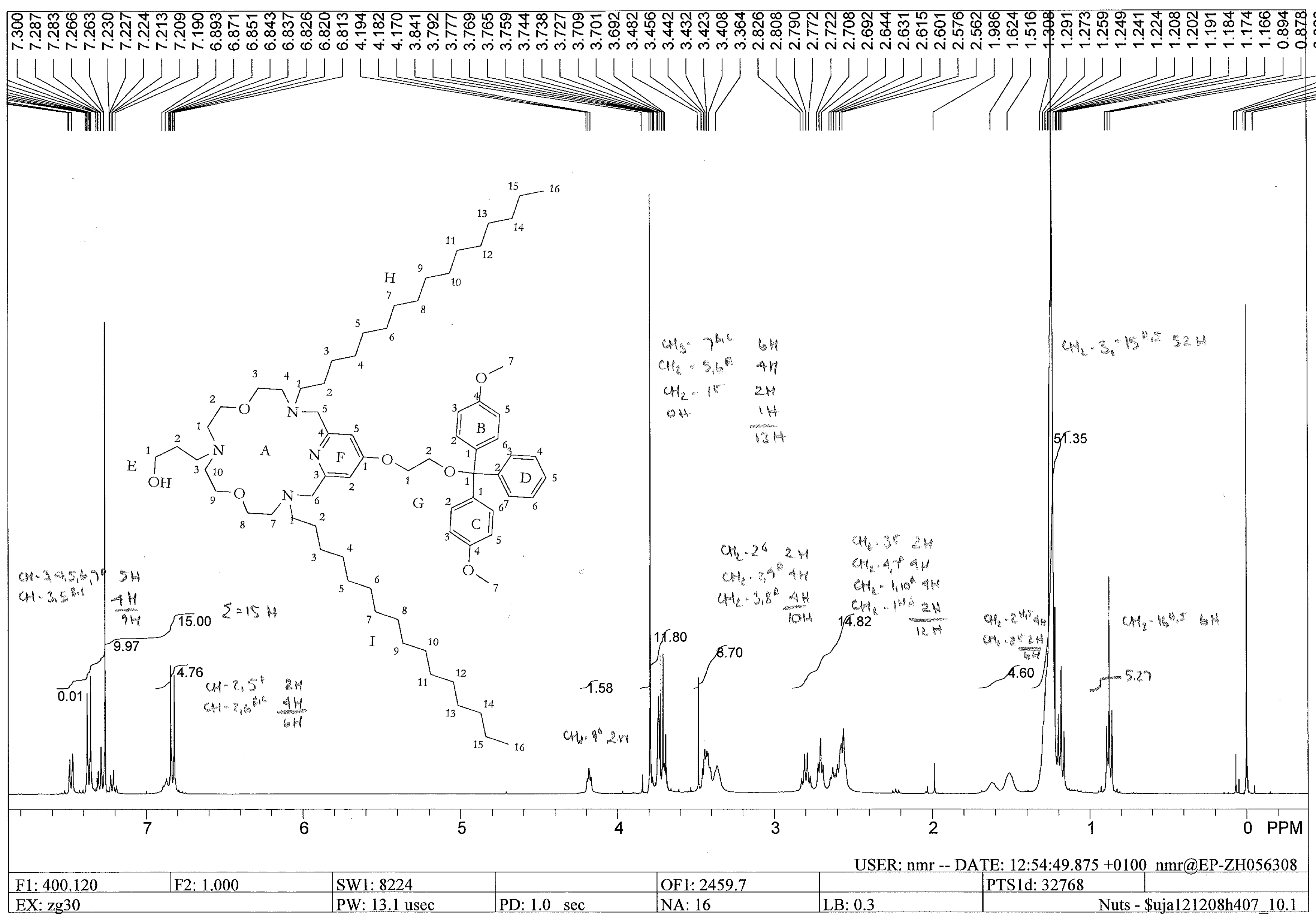


13C-NMR spectrum of 5 (101 MHz, CDCl_3_, 25 °C)

**
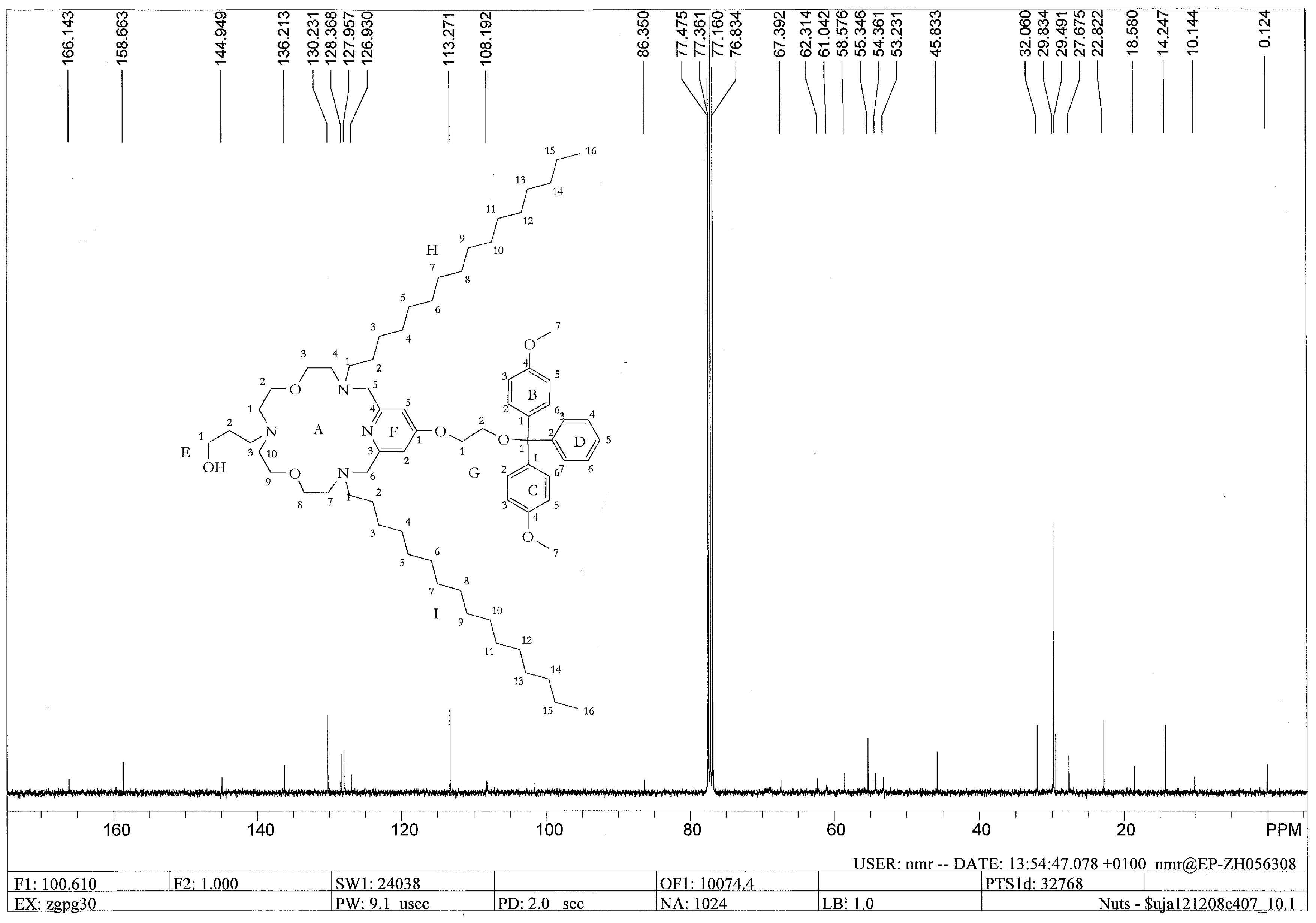
**

Supplemental data and discussion

Thermal denaturation data

The T_m_ values for the two LiNA pairs were determined in buffer in absence of liposomes, to verify their ability to hybridize. Also the T_m_ for pairs of LiNAs with a purchased reference strand with the same 17 nt „zipper“ sequences but without anchors (Ref-A and Ref-A‘) were obtained. For melting points of LiNA duplexes in buffer, hydrophobic interactions between the four C_16_-hydrocarbon anchors in the duplex increase melting temperatures substantially (ΔTm = +20-23 °C) compared to the reference duplex as well as the LiNA/DNA pairs. The T_m_ of the LiNA/DNA duplexes gives an estimate of T_m_ for liposome anchored LiNA-duplexes, where the C16-moieties of each strand are unable to interact as they are embedded in different bilayers.

**Table S2.** Thermal denaturation of LiNAs and reference strands.

| ds(DNA) pair  T_m_ (°C)^[a]^ | **LiNA-1** | **LiNA-3** | **Ref-A** |
| --- | --- | --- | --- |
| **LiNA-2** | 78.3 ± 0.7^[b]^ | n.d. | 56.2 ± 0.5 |
| **LiNA-4** | n.d. | 76.4 ± 0.6 | 57.7 ± 0.4 |
| **Ref-A’** | 55.9 ± 0.3 | 56.9 ± 3 | 55.3 ± 0.4 |

^[a]^ measured at 1 µM of each oligonucleotide in HBS.

^[b]^ average of five ramps between 20 and 90 °C and duplicate samples. In parenthesis the standard deviation to the average is given.

CD spectra

**Figure S1.** Selected CD spectra. 1 µM DNA/DNA, DNA/LiNA or LiNA/LiNA duplexes in HBS buffer. 22 °C.

Nanoparticle tracking data

**Figure S3.** Example of size-distribution envelope. Nanoparticle tracking analysis (NTA) data for a SRB-labeled population. SD = standard deviation of the distribution histogram (bin width 2 nm). Average diameter 134.4 ± 6.0 (5 repeats, 2 independent samples, ± total error)

The size distribution during the fusion experiment was monitored by subjecting aliquots of a fusion experiment (unlabeled liposomes with LiNA-1 and LiNA-2, respectively, 1:1 ratio, ~65 LiNAs pr. Liposome). After one minute a very broad size-distribution is observed with a mode diameter above 200 nm, but at 30 min, the distribution narrowed again, indicating that aggregates of three or more liposomes might be formed transiently at the beginning of the fusion process (**Figure S2**). Given the LiNA copy number on the liposome surface, it seems logical that initially any liposome may interact with multiple liposomes bearing the complementary strand. At this stage, some intervesicle contacts will recruit more LiNAs than others, leading to fusion between some liposomes but release of others. When following the mean diameter of liposomes in a fusion experiment over time (**Figure S3**), the resulting trend is congruent with the above consideration. Between 1-5 min, the average diameter already recedes, which matches the CM experiment insofar as the rate of fluorescence increase slows and that the CM after 5 min is >90% of the value at 30 min already.

**Figure S4**. Size distribution envelopes of fusion samples at different time-points.

**Figure S5.** Mean diameter of liposomes recorded from samples taken during a fusion experiment at 50 ° C. .Error bars: Total error between mean diameters obtained for duplicate samples

Additional data for content mixing assay

The assay is based on the self-quenching properties of 20 mM SRB entrapped in vesicles and has previously been used to measure content mixing.^3,4^ The fluorescence of an SRB-filled vesicle suspension is inversely proportional to the concentration of entrapped SRB. Upon content mixing with unlabeled liposomes - or leakage into the outer medium - the dye is diluted, which leads to an increase in fluorescence (Apparent CM). The measured fluorescence increase is thus based on both content mixing and leakage and must be compared to an experiment where the entrapped [SRB] is the same in all liposomes and any fluorescence increase must stem from leakage (Main manuscript, Figure 3). Figure S5 gives representative fluorescence data for both apparent CM of the two LiNA setups to compare with controls with non-complementary LiNA-1 on both populations or no LiNAs at all. In both controls the signal increase must be due to passive processes, and the comparison shows that the presence of (non-complementary) LiNAs reduces these passive processes when no fusion is involved.

**Figure S6:** Apparent CM data for LiNA-1/2 (X^E^), LiNA-3/4 (X^E^-P3), and controls.

Initial fusion rate data

Asymptotic linear fits to the fusion yield data for the current (X^E^) and the previously studied (X^N^) anchors.

**Figure S6.** Initial fusion rates of the dipalmitylated crown-ether conjugated LiNA pairs (top panels) versus the rates observed for the previously reported aminopropane conjugated LiNAs.

1 Cavaluzzi, M. J. & Borer, P. N. Revised UV extinction coefficients for nucleoside-5'-monophosphates and unpaired DNA and RNA. *Nucleic Acids Res* **32**, e13, doi:10.1093/nar/gnh015 (2004).

2 Rohr, K. & Vogel, S. Polyaza crown ethers as non-nucleosidic building blocks in DNA conjugates: synthesis and remarkable stabilization of dsDNA. *ChemBioChem* **7**, 463-470, doi:10.1002/cbic.200500392 (2006).

3 Lygina, A. S., Meyenberg, K., Jahn, R. & Diederichsen, U. Transmembrane domain peptide/peptide nucleic acid hybrid as a model of a SNARE protein in vesicle fusion. *Angew Chem Int Ed Engl* **50**, 8597-8601, doi:10.1002/anie.201101951 (2011).

4 Zheng, T. *et al.* Controlling the rate of coiled coil driven membrane fusion. *Chemical Communications* **49**, 3649-3651, doi:10.1039/C3CC38926J (2013).
